# Inclusion of MUC1 (Ma695) in a panel of immunohistochemical markers is useful for distinguishing between endocervical and endometrial mucinous adenocarcinoma*

**DOI:** 10.1186/1472-6890-6-1

**Published:** 2006-01-12

**Authors:** Thaer Khoury, Dongfeng Tan, Jianmin Wang, Marilyn Intengan, Jun Yang, Sadir Alrawi, Peisha Yan, James C Byrd

**Affiliations:** 1 Department of Pathology, Roswell Park Cancer Institute, State University of New York at Buffalo, NY; 2 Department of Pathology, University of Texas Health Science Center at Houston, TX; 3 Department of epidemiology, Roswell Park Cancer Institute, NY; 4 University of Texas MD Anderson Cancer Center, Houston, TX

## Abstract

**Background:**

Distinguishing endocervical adenocarcinoma (ECA) from endometrial mucinous adenocarcinoma (EMMA) is clinically significant in view of the differences in their management and prognosis. In this study, we used a panel of tumor markers to determine their ability to distinguish between primary endocervical adenocarcinoma and primary endometrial mucinous adenocarcinoma.

**Methods:**

Immunohistochemistry using monoclonal antibodies to MUC1 (Ma695), p16, estrogen receptor (ER), progesterone receptor (PR), and vimentin, was performed to examine 32 cases, including 18 EMMAs and 14 ECAs. For MUC1, cases were scored based on the percentage of staining pattern, apical, apical and cytoplasmic (A/C), or negative. For p16, cases were scored based on the percentage of cells stained. For the rest of the antibodies, semiquantitative scoring system was carried out.

**Results:**

For MUC1, majority of EMMA (14 of 18 cases, 78%) showed A/C staining, whereas only few ECA (2 of 14, 14%) were positive. The difference of MUC1 expression in the two groups of malignancy was statistically significant (*p < 0.001*). Staining for p16 was positive in 10 of 14 (71%) ECA and 4 of 18 (22%) EMMA. Estrogen receptor was positive in 3 of 14 (21%) ECA and 17 of 18 (94%) EMMA. Progesterone receptor was positive in 3 of 14 (21%) ECA and 16 of 18 (89%) EMMA. Vimentin was positive in 1 of 14 (7%) ECA, and 9 of 18 (50%) EMA, with median and range of 0 (0–6), and 1.5 (0–9) respectively.

**Conclusion:**

A panel of immunohistochemical markers including MUC1, p16, ER, PR, and vimentin is recommended, when there is morphological and clinical doubt as to the primary site of endocervical or endometrial origin.

## Background

Distinguishing endocervical adenocarcinoma from its endometrial counterpart is clinically significant in view of their differences in management and prognosis. While the treatment of endometrial carcinoma starts with surgical staging and intraoperative assessment of the grade and extent of tumor in the uterus, primary endocervical carcinoma is treated by an initial radical hysterectomy and pelvic lymphadenectomy with or without adjuvant radiation [1,2].

Morphologic distinction of the two gynecologic neoplasms can be difficult when the tumor involves the lower uterine segment or upper endocervix, when the adenocarcinoma is present in both components of a fractional dilation and curettage (D&C) specimen, or when there is no in-situ component, cervical glandular intraepithelial (CGIN), cervical intraepithelial neoplasia (CIN), or endometrial hyperplasia [3,4]. This distinction could be even more difficult in the mucinous subtype in which stroma is absent, one of the features that suggests an endometrial origin. Several studies have reported the use of immunohistochemical analysis with monoclonal antibodies to distinguish between adenocarcinomas of endometrial and endocervical origin [3-17].

Mucins are high-molecular-weight (>200 kDa) glycoproteins with oligosaccharides attached to an apomucin protein backbone (core peptide) by O-glycosidic linkage [18,19]. They are mainly synthesized by epithelial cells and can be classified as secretory (gel-forming) and membrane-associated forms. MUC1 is a transmembrane protein with a large extracellular tandem repeat domain and can be found on the apical surface of almost all glandular and ductal epithelial cells. There are also soluble, secreted forms of MUC1 mucin, generated by proteolysis and/or alternative mRNA splicing [20]. Aberrant *de novo *expression, overexpression, or altered glycosylation of MUC1 has been demonstrated in several human malignancies [21]. Though the physiological role of MUC1 in the female genital tract and its expression in endometrial hyperplasia and carcinoma have been investigated [22-28], the utility of MUC1 in differential diagnosis of endocervical adenocarcinoma and endometrial mucinous adenocarcinoma has been little explored.

The objectives of this study were to evaluate a panel of monoclonal antibodies (MUC1, p16, ER, PR, and vimentin), and assess their diagnostic value in distinguishing between primary endocervical adenocarcinoma (ECA), and primary endometrial mucinous adenocarcinoma (EMMA).

## Methods

### Cases

Formalin-fixed, paraffin-embedded tissue blocks containing mucinous adenocarcinomas of known origin, endocervix, and endometrium were obtained from the surgical pathology files at Roswell Park Cancer Institute, Buffalo, New York. Only primary endocervical and endometrial adenocarcinomas from hysterectomy or conization specimens with negative hysteroscopy were included in this study. Small biopsy specimens were excluded from the study. There were 18 endometrial mucinous adenocarcinoma, and 14 endocervical adenocarcinomas of "typical" type, showing endometrioid and mucinous features.

### Immunohistochemical staining

Using the avidin-biotin complex technique, slides were stained with the following monoclonal antibodies whose main characteristics are summarized in Table 1.

Monoclonal antibody Ma695 (purchased from Novocastra, Newcastle, UK) recognizes a sialylated carbohydrate antigen on MUC1 mucin [29]. For MUC1 staining, Ma695 was used at a 1:100 dilution with high temperature unmasking technique, with colonic mucosa as a positive control. Staining for p16 used antibody E6H4 (DAKO, Carpinteria, CA) at 1:25 dilution, with TRS retrieval and high grade squamous dysplasia as a positive control. Staining conditions for vimentin, estrogen receptor (ER), and progesterone receptor (PR) are summarized in Table 1. Appropriate positive and negative controls were performed.

### Scoring of immunostaining

Due to the heterogeneity of Immunostaining, two blocks from each case were stained and scored combined. The immunoreactive score for MUC1 was the sum-total of the percentage of each staining pattern, "purely apical", or "combined apical/cytoplasmic" (A/C). For example, if MUC1 showed "pure apical staining" in 40% of tumor cells, "combined A/C staining" in 40% of tumor cells, and 20% of tumor cells did not stain, the scores would be 40 apical, 40 A/C, and 20 negative. These scores were statistically analyzed in a 2 × 4 table. Each staining pattern was considered positive if it was present in >20% of the tumor. Staining for p16 was scored depending on the percentage of positively staining tumor cells, where p16 was considered diffusely positive when expressed in >50% of tumor cells. A semiquantitative scoring system was applied for ER, PR, and vimentin following the German Immunohistochemical Scoring System [30] in which the final immunoreactive score equaled the product of the percentage of positive cells times the average staining intensity (4). Percentage of positive cells was graded as follows: 0 = negative, 1 = up to 10% positive cells, 2 = 11 to 50%, 3 = 51 to 80%, 4 = >80%. Staining intensity of 0 = negative, 1 = weakly positive, 2 = moderately positive, 3 = strongly positive. A combination of 3 or greater was considered positive for ER, PR and vimentin.

### Statistical analysis

Descriptive analysis was performed to evaluate the frequencies and distributions of the analytic variables. Given the fact that most of the variables were not normally distributed, we applied Mann-Whitney U-test, a nonparametric analysis technique, to examine the median difference of each immunohistochemical biomarker between patients with endocervical and endometrial mucinous tumors. We also used Chi-square to test the difference of each immunohistochemical biomarker between these two groups of patients where the biomarker was classified as positive or negative for each patient. Medians, ranges of values, and p values were reported. All tests were 2-sided and the significance level was 0.05. All analyses were performed using SPSS statistical software (SPSS, Inc., Chicago, IL).

## Results

**Figure 1:**
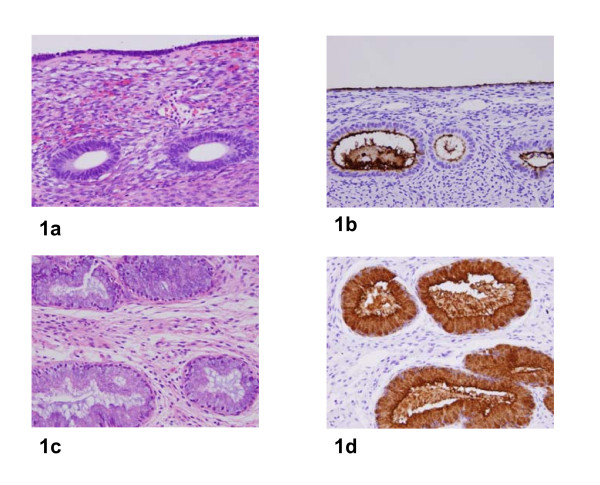
A, Normal post-menopausal endometrium (H&E, 20x); B, MUC1 immunostain in normal postmenopausal endometrium showing “pure apical” staining pattern (x20); C, normal endocervical glands (H&E, 20x); D, MUC1 immunostain in normal endocervical glands showing A/C staining pattern, (x20).

**Figure 2:**
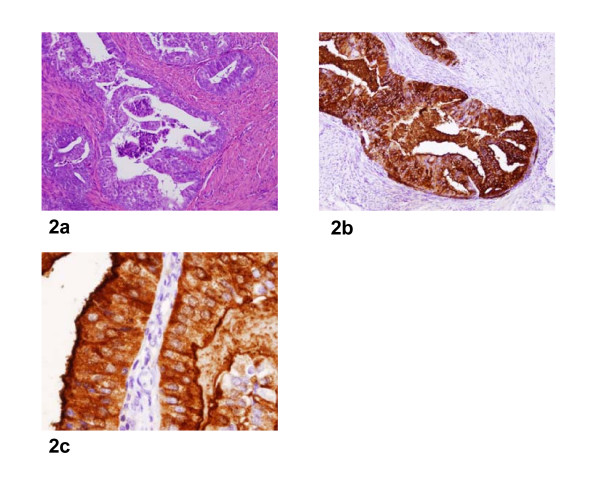
A, Endometrial mucinous adenocarcinoma (H&E, x10); B, MUC1 immunostain in endometrial mucinous adenocarcinoma showing A/C staining pattern (x10); C, MUC1 immunostain in endometrial mucinous adenocarcinoma showing A/C staining pattern (x40).

**Figure 3:**
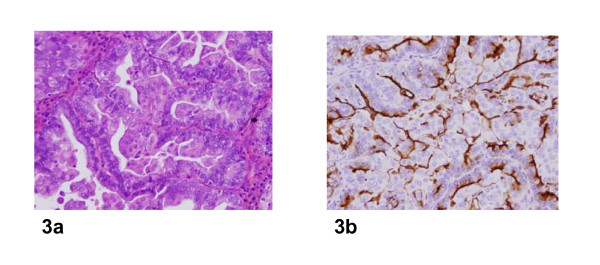
A, Endocervical adenocarcinoma (H&E, x20); B, MUC1 immunostain in endocervical adenocarcinoma showing apical staining pattern (x20).

MUC1 had two different expression patterns, "purely apical", similar to that in normal endometrium (Fig. 1A, 1B), and combined A/C, as seen in normal endocervical glands (Fig. 1C, 1D). Apical staining was recorded, when distinct membranous staining facing the lumen was present. Cytoplasmic staining was considered present when complete circumferential cytoplasmic staining including the subnuclear area was evident. Table 2 shows the results of MUC1 staining. Apical and cytoplasmic MUC1 was seen in 14 of 18 cases (78%) of EMMA, but only 2 of 14 cases (14%) of ECA (p < 0.001). In EMMA, the median percentage of cells with apical and cytoplasmic MUC1 was 70% (range 0–100), while in ECA the median was 0% (range 0–80). Apical and cytoplasmic expression of MUC1 in EMMA is exemplified in Figure 2. Purely apical MUC1, in contrast, was more common in ECA (8 of 14 = 57% of cases) than in EMMA (7 of 18 = 39% of cases). In ECA, the median percentage of cells with apical MUC1 was 35% (range 0–100), while in EMMA the median was 15% (range 0–90). Apical staining for MUC1 in ECA is illustrated in Figure 3. The median percentage of cells negative for MUC1 was higher for ECA (35%, range 0–100) than for EMMA (0%, range 0–90). There was no correlation between presence of intracytoplasmic mucin and MUC1 staining pattern. In general, while EMMA showed an apical/cytoplasmic pattern, ECA was either negative or purely apical.

The results of expression of estrogen receptor, progesterone receptor, and vimentin are shown in Table 3. Using >3 as a cutoff, ER was positive in 3 of 14 (21%) ECA, and 17 of 18 (94%) EMMA, (*p < 0.001*), with median and range of 0 (0–100), and 7.5 (2–12) respectively (*p < 0.001*). Progesterone receptor was positive in 3 of 14 (21%) ECA, and 16 of 18 (89%) EMMA, (*p < 0.001*), with median and range of 0 (0–12), and 9 (1–12) respectively, (*p < 0.001*). Endometrial adenocarcinoma had positive staining for ER and PR far more than ECA. No statistically significant correlation was seen between any variable of MUC1 and ER or PR.

A cutoff >1, weak staining in 1–10% of the cells, was used to evaluate the results of vimentin. Vimentin was positive in 1 of 14 (7%) ECA, and 9 of 18 (50%) EMMA, (*p = 0.01*), with median and range of 0 (0–2), and 1.5 (0–9) respectively, (*p = 0.002*). Using higher cutoff, vimentin lost its significant value in distinguishing between ECA and EMMA.

Table 4 shows the results of p16. In most cases, p16 staining was of both nuclear and cytoplasmic staining pattern. Using >50% as a cutoff, p16 was positive in 10 of 14 (71%) ECA, and 4 of 18 (22%) EMMA, (*p = 0.005*), with median and range of 85 (0–100), and 30 (0–100) respectively (*p = 0.007*). In general, p16 was diffuse in ECA and patchy in EMMA.

## Discussion

The results of this study suggested that a panel of monoclonal antibodies directed against MUC1, p16, ER, PR, and vimentin helped distinguish between primary ECA and primary EMMA. While primary ECA showed diffusely positive p16, negative or apical MUC1, and negative ER/PR, and vimentin, primary EMMA revealed either negative or focally positive p16, A/C MUC1, and positive ER/PR, and vimentin. Due to the difficulties in distinguishing the primary site of the tumor using H&E alone, and since preoperative assessing of D&C specimen is crucial in planning the patient management, this panel could be helpful in such cases.

MUC1 (Ma695) has been studied in normal cycling endometrium. It was consistently expressed in the normal endometrium, following a cyclical pattern: "apical membrane staining" in early and mid-proliferative endometrium; "purely cytoplasmic staining" in late proliferative endometrium; and "cytoplasmic staining with intraluminal secretions" in secretory endometrium [22]. It has been suggested that mucin was hormonally regulated. It was expressed with increased abundance in the secretory phase of the menstrual cycle in rabbits, revealing an upregulation function for progesterone [28]. Staining of MUC1 by Ma695 in postmenopausal endometrium has not been explored. We found that MUC1 expression in normal postmenopausal endometrium was either negative or apical. Absence of cytoplasmic expression could be explained by below-threshold progesterone level in this population group.

MUC1 expression was tested in endometrial carcinomas. They were of "purely apical pattern", "purely cytoplasmic pattern" or negative. Apical MUC1 positivity was statistically more frequent in endometrioid carcinomas compared with carcinomas of non-endometrioid type [22]. In our study, mucinous adenocarcinoma was the only subtype studied. This may at least in part explain why most cases had A/C staining.

While upregulation of progesterone increased cytoplasmic expression of MUC1 in normal cycling endometrium [28], negative MUC1 was significantly related to PR expression and marginally correlated with loss of ER in endometrial adenocarcinoma (EMA) [22]. In our study, there was no correlation between any of MUC1 variables and ER or PR. The small number of cases, case selection being all of mucinous subtype, or both could explain these results.

MUC1 expression in the non-neoplastic cervical epithelium has been studied. It was unaffected by ovarian steroids, confirming tissue-specific regulation of MUC1 in the lower reproductive tract [28]. However, this notion has not been tested in endocervical adenocarcinoma. In our study, normal endocervical glands predominantly expressed A/C MUC1, while the majority of ECAs were either negative or apical. In ECA, no statistically significant correlation between any variable of MUC1 and ER or PR was seen. While two cases had diffuse A/C MUC1, none had ER or PR expression. We concluded that cytoplasmic expression of MUC1 in ECA was unlikely affected by hormonal expression in these neoplasms. However, due to the limited number of cases, more studies with larger number of cases are required to confirm this assumption.

p16, cyclin-dependent kinase-4 inhibitor (CDK4-I), is the product of the ink 4a gene and specifically binds to cyclin D-cdk4/6 complexes to control the cell cycle at the G1-S interphase. Expression of p16 has been shown in preinvasive, high-grade, cervical squamous lesions and in low-grade lesions associated with high-risk human papillomavirus (HPV) types [31-34]. p16 overexpression in HPV-associated lesions is probably caused by inactivation of the retinoblastoma protein, Rb, by the E7 HPV oncoprotein, which acts as a p16 transcript repressor [35].

Positive p16 staining of cervical adenocarcinoma in situ and adenocarcinoma has been noted [31-34,36]. It has been used to distinguish between ECA and endometrial adenocarcinoma. In these studies, p16 was diffusely positive in ECA and negative or focally positive in EMA. However, few ECAs were negative, and few EMAs were diffusely positive [16,17].

Although McCluggage *et al*. [16] studied p16 expression in endometrioid endometrial adenocarcinoma, and we studied mucinous adenocarcinoma, diffuse p16 (>50%) was seen in 28% and 22% of respectively. We concluded that regardless of tumor type, p16 could be diffusely expressed in a relatively small percentage of EMAs.

It was unclear why diffuse p16 expression was present in EMMA. One possibility was that p16 positivity was a result of HPV-independent mechanisms. This theory was supported by Ansari-Lari et al. [17], who tested HPV in situ hybridization and p16 immunostaining in 24 EMAs. They found that while HPV was not detected in any EMA, moderate or strong p16 staining in = 50% of tumor cells was seen in 25% of the cases. Previous studies, however, have identified HPV subtypes in a minority of endometrial adenocarcinomas [37-39]. Patchy p16 positivity may be associated with infection by low risk HPV subtypes, because this pattern of staining may be found in low-grade squamous intraepithelial lesions associated with low-risk HPV subtypes.

We found, as others did [16], that few ECAs were completely negative (n = 2), and (n = 1) respectively. The reasons for these results were also unclear. It was either due to technical failure or as a result of HPV-independent mechanisms [16]. In another study, HPV DNA was detected in 14 of 18 positive ECAs, and p16 was diffusely expressed in all ECAs [17]. The authors concluded that negative HPV DNA detection was due to technical failure of the in situ hybridization and PCR reaction.

Reviewing the comprehensive immunoprofile of the 32 cases showed that two or more "aberrant" antibody expressions of ER, PR, vimentin, and p16 were seen in three ECAs (#5, 6, and 8), and one EMMA (#22) Table 5. ER+/ PR+ immunoprofile was seen in case#5. A hysterectomy specimen was examined. A primary endocervical adenocarcinoma with no endometrial involvement was seen. ER+/PR+ and focal p16 immunoprofile was seen in case #6. It was a mucinous adenocarcinoma involving an endocervical polyp. No endometrial involvement was present in hysteroscopy. A PR+/p16- immunoprofile was seen in case #8. A hysterectomy specimen was examined revealing an endocervical adenocarcinoma with no endometrial involvement. The immnoprofile of these cases was not consistent with either ECA or EMMA, however, cytoplasmic expression of MUC1 (0, 20, and 0 respectively) was consistent with ECA. In case #23 (EMMA), "aberrant" negative vimentin and PR expression was seen. A hysterectomy specimen examination revealed EMMA with no endocervical involvement. Cytoplasmic MUC1 was present in 80% of the tumor cells, which made the tumor more consistent with endometrial primary.

A recent study investigated whether the immunophenotype of mucinous carcinoma of the endometrium and endometrioid carcinoma of the cervix was more dependent on the site of origin or the differentiation of the neoplasm [3]. It has been found that ER (clone CC4–5) staining was more dependent on the site of origin, being more common in endometrial than endocervical carcinoma, whereas vimentin positivity is more dependent on the pattern of differentiation, being more common in endometrioid than mucinous neoplasms. They concluded that if a tumor exhibited strong positive staining with vimentin and ER, it was almost certainly of endometrial origin. In our study, mucinous type of endometrial carcinoma was studied. This may explain the weak vimentin positivity. Estrogen receptor and PR had a very good value in distinguishing between primary ECA and primary EMMA.

## Conclusion

Although several groups have investigated the use of immunohistochemical markers to distinguish between endocervical and endometrial adenocarcinoma, the findings were inconclusive and sometimes conflicting. The reasons behind these variable results may be multifactoral, including differences in clones or retrieval processes used; difference in the selected cases for example mucinous versus endometrioid adenocarcinoma; or the small size of tested specimens. In our study, mucinous subtype of endometrial carcinoma was studied; all small biopsies were excluded; the tumor origin was surgically verified by examining the gross specimen; new antibody (MUC1); and new monoclonal antibody clones for ER and PR were used.

For practical purposes, if two or more immunostains have to be combined, any two markers could be significantly useful in distinguishing ECA from EMMA. However, combining MUC1 with p63, ER, PR, or both ER/PR could give the best results as illustrated in Table 6.

We found that a panel of immunohistochemical markers consisting of ER, PR, p16, and MUC1 reliably distinguishes between primary ECA and primary EMMA. Vimentin could be added to this panel when relatively larger specimen is represented. Because surgical modalities may differ, such a panel should be routinely used before definitive surgery when there is morphological and clinical doubt as to the primary site of endocervical or endometrial adenocarcinoma origin.

## Note

*Presented in part at the annual meeting of the American Society of Clinical Pathologists, San Antonio, TX, October, 2004.

## Pre-publication history

The pre-publication history for this paper can be accessed here:



## Figures and Tables

**Table 1:** Characteristics of antibodies used for evaluation.

**Antibody**	**Clone**	**Source**	**Antigen retrieval**	**Dilution**
MUC1	Ma695	Novocastra, Newcastle, UK	High temperature	1:100
P16	E6H4	DAKO, Carpinteria, CA	TRS	1:25
Vim	Vim3B4	DAKO, Carpinteria, CA	TRS	1:1000
ER	1D5	DAKO, Carpinteria, CA	TRS/Vector	1:100
PR	PgR636	DAKO, Carpinteria, CA	TRS/Vector	1:200

**Table 2:** MUC1 expression in endocervical and endometrial adenocarcinomas

	**Median (range)**			+ **Cases/Total (%)^1^**		
	**ECA**	**EMMA**	**(*p-value*)**	**ECA**	**EMMA**	**(*p-value*)**

**Apical**	35 (0–100)	15 (0–90)	*NS*	8/14 (57%)	7/18 (39%)	NS
**A/C**	0 (0–80)	70 (0–100)	*<0.001*	2/14 (14%)	14/18 (78%)	<0.001
**Negative**	35 (0–100)	0 (0–90)	*0.01*	7/14 (50%)	2/18 (11%)	0.01

ECA, endocervical adenocarcinoma; EMMA, endometrial mucinous adenocarcinoma; A/C, apical/cytoplasmic; NS, not significant. ^1 ^totals are >100% because different areas of tumors differed in staining pattern.

**Table 3:** Immunostaining results of other markers studied.

		**ECA**	**EMMA**	***p-value***
**ER**	Score 0–3	11/14 (79%)	1/18 (6%)	*<0.001*
	Score 4–12	3/14 (21%)	17/18 (94%)	
	Median (Range)	0 (0–12)	7.5 (2–12)	*<0.001*
**PR**	Score 0–3	11/14 (79%)	2/18 (11%)	*<0.001*
	Score 4–12	3/14 (21%)	16/18 (89%)	
	Median (Range)	0 (0–12)	9 (1–12)	*<0.001*
**Vim**	Score 0–3	13/14 (93%)	9/18 (50%)	*0.01*
	Score 4–12	1/14 (7%)	9/18 (50%)	
	Median (Range)	0 (0–2)	1.5 (0–9)	*0.002*

ECA, endocervical adenocarcinoma; EMMA, endometrial mucinous adenocarcinoma; NS, not significant.

**Table 4:** p16 expression in endocervical and endometrial adenocarcinomas.

	**ECA**	**EMMA**	***p-value***
**0**	2	5	
**1–10**	1	1	
**11–50**	1	8	
**Total score (0–50)**	4/14 (29%)	14/18 (78%)	
**51–80**	3	3	
**81–100**	7	1	
**Total score (51–100)**	10/14 (71%)	4/18 (22%)	*0.005*
**Median (Range)**	85 (0–100)	30 (0–100)	*0.007*

ECA, endocervical adenocarcinoma; EMMA, endometrial mucinous adenocarcinoma.

**Table 5:** Comprehensive list of all 33 cases showing the immunohistochemical profile of each case.

**Case#**	**Tumor site**	**MUC1 (%)**			**Vim**	**ER**	**PR**	**P16 (%)**
		**Apical**	**A/C**	**N**				

1	EC	100	0	0	0	0	0	0
2	EC	0	0	100	0	0	0	100
3	EC	0	0	100	0	0	0	100
4	EC	80	20	0	0	0	0	90
5	EC	50	0	50	0	6	12	90
6	EC	70	20	10	0	12	4	10
7	EC	30	0	70	0	4	0	80
8	EC	100	0	0	0	0	6	0
9	EC	60	20	20	0	0	0	100
10	EC	0	0	100	0	2	0	80
11	EC	0	0	100	0	1	0	80
12	EC	0	0	100	0	0	0	50
13	EC	0	80	20	0	0	0	100
14	EC	40	60	0	2	0	0	100
15	EM	50	40	10	2	4	2	40
16	EM	90	10	0	0	6	12	0
17	EM	10	90	0	4	9	9	30
18	EM	70	10	20	0	4	9	30
19	EM	0	100	0	6	6	12	70
20	EM	50	40	10	2	12	12	0
21	EM	0	90	10	0	2	12	60
22	EM	20	80	0	0	12	1	100
23	EM	0	100	0	0	12	12	0
24	EM	0	100	0	9	4	9	0
25	EM	0	100	0	9	12	6	20
26	EM	10	90	0	4	12	12	70
27	EM	10	0	90	1	12	9	30
28	EM	80	20	0	0	6	6	10
29	EM	0	60	40	0	6	9	0
30	EM	40	50	10	4	6	9	40
31	EM	60	40	0	2	12	9	40
32	EM	20	80	0	9	12	9	40

EC : endocervix, EM, endometrial.

**Table 6:** Panel expressions of combining at least two immunostains in endocervical and endometrial adenocarcinomas.

		**ECA**	**EMMA**	*p-value*
MUC1* & P16	MUC1+, P16-	0 (0%)	10 (100%)	*0.001*
	MUC1-, P16+	8 (100%)	0 (0%)	
**MUC1 & VIM**	MUC1+, VIM+	1 (7.7%)	9 (69.2%)	*0.004*
	MUC1-, VIM-	12 (92.3%)	4 (30.8%)	
**MUC1 & ER**	MUC1+, ER+	0 (0%)	13 (100%)	*0.001*
	MUC1-, ER-	9 (100%)	0 (0%)	
**MUC1 & PR**	MUC1+, PR+	0 (0%)	12 (72.2%)	*0.001*
	MUC1-, PR-	9 (100%)	0 (27.8%)	
**P16 & VIM**	P16-, VIM+	0 (0%)	7 (77.8%)	*0.002*
	P16+, VIM-	9 (100%)	2 (22.2%)	
**P16 & ER**	P16-, ER+	1 (11.1%)	14 (93.3%)	*0.001*
	P16+, ER-	8 (88.9%)	1 (6.7%)	
**P16 & PR**	P16-, PR+	2 (18.2%)	13 (92.9%)	*0.001*
	P16+, PR-	9 (81.8%)	1 (7.1%)	
**VIM & ER**	VIM+, ER+	0 (0%)	9 (90%)	*0.001*
	VIM-, ER-	10 (100%)	1 (10%)	
**VIM & PR**	VIM+, PR+	0 (0%)	8 (88.9%)	*0.001*
	VIM-, PR-	10 (100%)	1 (11.1%)	
**ER & PR**	ER+, PR+	2 (16.7%)	15 (100%)	*0.001*
	ER-, PR-	10 (83.3%)	0 (0%)	
**MUC1, ER & PR**	MUC1+, ER+, PR+	0 (0%)	11 (100%)	*0.001*
	MUC1-, ER-, PR-	8 (100%)	0 (0%)	

ECA, endocervical adenocarcinoma; EMMA, endometrial mucinous adenocarcinoma. * Apical and cytoplasmic MUC1 expression.

## References

[B1] Berek JS, Hacker NV, Lippincott Williams, Wilkins (2000). cervical cancer, and uterine cancer. Practical Gynecologic Oncology.

[B2] Hoskins WJ, Perez CA, Young RC, Lippincott JB (2005). uterine cervix, and corpus: epithelial tumors. Principles and Practice of Gynecologic Oncology.

[B3] Kamoi S, AlJuboury MI, Akin M, Silverberg SG (2002). Immunohistochemical staining in the distinction between primary endometrial and endocervical adenocarcinomas: Another viewpoint. Int J Gyn Pathol.

[B4] McCluggage WG, Sumathi VP, McBride HA, Patterson A (2002). A panel of immunohistochemical stains including carcinoembryonic antigen, vimentin, and estrogen receptor aids in the distinction between primary endometrial and endocervical adenocarcinomas. Int J Gynecol Pathol.

[B5] Zaino RJ (2002). The fruits of our labors: distinguishing endometrial from endocervical adenocarcinoma. Int J Gynecol Pathol.

[B6] Wahlstrom T, Lindgren J, Korhonen M, Seppala M (1979). Distinction between endocervical and endometrial adenocarcinoma with immunoperoxidase staining of carcinoembryonic antigen in routine histological specimens. Lancet.

[B7] Cohen C, Shulman G, Budgeon L (1982). Endocervical and endometrial adenocarcinoma: an immunoperoxidase and immunohistochemical study. Am J Surg Pathol.

[B8] Dabbs DJ, Geisinger KR, Norris HT (1986). Intermediate filaments in endometrial and endocervical carcinomas. The diagnostic utility of vimentin patterns. Am J Surg Pathol.

[B9] Dabbs D, Geisinger K (1993). Selective applications of immunohistochemistry in gynecological neoplasms. Pathol Annu.

[B10] Dabbs D, Sturtz K, Zaino R (1996). The immunohistochemical discrimination of endometrioid adenocarcinomas. Hum Pathol.

[B11] Toda T, Sadi AM, Egawa H, Atari E, Qureshi B, Nagai Y (1998). Affinity of four lectins for endocervical and endometrial non-neoplastic and neoplastic glandular epithelium. Histopathology.

[B12] Staebler A, Sherman ME, Ronnett BM (2001). Hormone receptor immunohistochemistry (IHC) and HPV in situ hybridization (ISH) are useful for distinguishing between primary endocervical and endometrial adenocarcinomas. Mod Pathol.

[B13] Castrillon D, Lee K, Nucci M (2002). Distinction between endometrial and endocervical adenocarcinoma: an immunohistochemical study. Int J Gynecol Pathol.

[B14] Maes G, Fleuren GJ, Bara J, Nap M (1988). The distinction of mucins, carcinoembryonic antigen, and mucus-associated antigens in endocervical and endometrial adenocarcinomas. Int J Gynecol Pathol.

[B15] Kudo R, Sasano H, Koizumi M, Orenstein JM, Silverberg SG (1990). Immunohistochemical comparison of new monoclonal antibody 1C5 and carcinoembryonic antigen in the differential diagnosis of adenocarcinoma of the uterine cervix. Int J Gynecol Pathol.

[B16] McCluggage WG, Jenkins D (2003). p16 immunoreactivity may assist in the distinction between endometrial and endocervical adenocarcinoma. Int J Gynecol Pathol.

[B17] Ansari-Lari MA, Staebler A, Zaino RJ, Shah KV, Ronnett BM (2004). Distinction of endocervical and endometrial adenocarcinomas: immunohistochemical p16 expression correlated with human papillomavirus (HPV) DNA detection. Am J Surg Pathol.

[B18] Dekker J, Rossen JW, Buller HA (2002). The MUC family: an obituary. Trends Biochem Sci.

[B19] Oniaux N, Escande F, Porchet N (2001). Structural organization and classification of the human mucin genes. Front Biosci.

[B20] Brayman M, Thathiah A, Carson DD (2004). MUC1: a multifunctional cell surface component of reproductive tissue epithelia. Reprod Biol Endocrinol.

[B21] Langner C, Ratschek M, Rehak P, Schips L, Zigeuner R (2004). Expression ofMUC1 (EMA) and E-cadherin in renal cell carcinoma: a systematic immunohistochemical analysis of 188 cases. Mod Pathol.

[B22] Sivridis E, Giatromanolaki A, Koukourakis MI, Georgiou L, Anastasiadis P (2002). Patterns of episialin/MUC1 expression in endometrial carcinomas and prognostic relevance. Histopathology.

[B23] Acosta AA, Elberger L, Borghi M (2000). Endometrial dating and determination of the window of implantation in healthy fertile women. Fertility & Sterility.

[B24] Carson DD, DeSouza MM, Kardon R, Zhao X, Lagow E, Julian J (1998). Mucin expression and function in the female reproductive tract. Human Reproduction Update.

[B25] Meseguer M, Pellicer A, Simon C (1998). MUC1 and endometrial receptivity. Molecular Human Reproduction.

[B26] Meseguer M, Aplin JD (2001). Human endometrial mucin MUC1 is up-regulated by progesterone and down-regulated in vitro by the human blastocyst. Biology of Reproduction.

[B27] Gipson IK (1997). Mucin genes expressed by human female reproductive tractepithelia. Biology of Reproduction.

[B28] Hewetson A, Chilton BS (1997). Molecular cloning and hormone-dependent expression of rabbit Muc1 in the cervix and uterus. Biol Reprod.

[B29] Price MR, Rye PD, Petrakou E (1998). Summary report on the ISOBM TD-4 Workshop: analysis of 56 monoclonal antibodies against the MUC1 mucin. Tumor Biol.

[B30] Remmele W, Schicketanz K-H (1993). Immunohistochemical determination of estrogen and progesterone receptor content in human breast cancer. Computer-assisted image analysis (QIC score) vs. subjective grading (IRS). Pathol Res Pract.

[B31] Keating JT, Cviko A, Reithdorf S, Riethdorf L, Quade BJ, Sun D, Duensing S, Sheets EE, Munger K, Crum CP (2001). Ki-67, cyclin E and p16 (INK 4) are complimentary surrogate biomarkers for human papilloma virus-related cervical neoplasia. Am J Surg Pathol.

[B32] Klaes R, Friedrich T, Spitkovsky D, Ridder R, Rudy W, Petry U, Dallenbach-Hellweg G, Schmidt D, von Knebel Doeberitz M (2001). Overexpression of p16 (INK 4A) as a specific marker for dysplastic and neoplastic epithelial cells of the cervix uteri. Int J Cancer.

[B33] Klaes R, Benner A, Friedrich T, Ridder R, Herrington S, Jenkins D, Kurman RJ, Schmidt D, Stoler M, von Knebel Doeberitz M (2001). p16 ink 4a immunohistochemistry improves interobserver agreement in the diagnosis of cervical intraepithelial neoplasia. J Pathol.

[B34] Murphy N, Bermingham N, Ring M (2001). Immunocytochemical analysis of p16 protein in cervical cancer and cervical intraepithelial neoplasia. J Pathol.

[B35] Ruas M, Peters G (1998). The p16 INK 4a/CDKN2A tumor suppressor and its relatives. Biochem Biophys Acta.

[B36] Cameron RI, Maxwell P, Jenkins D, MCluggage WG (2002). Immunohistochemical staining with MIB1, bcl-2 and p16 assists in the distinction of cervical glandular intraepithelial neoplasia from tubo-endometrial metaplasia, endometriosis and microglandular hyperplasia. Histopathology.

[B37] Semczuk A, Stenzel A, Baranowski W, Rozynska K, Cybulski M, Kostuch M, Jakowicki J, Wojcierowski J (2000). Detection of human papillomavirus types 16 and 18 in human neoplastic endometrium: lack of correlation with established prognostic factors. Oncol Reports.

[B38] Brewster WR, Monk BJ, Burger RA, Bergen S, Wilczynski SP (1999). Does human papillomavirus have a role in cancers of the uterine corpus?. Gynecol Oncol.

[B39] O'Leary JJ, Landers RJ, Crowley M, Healy I, O'Donovan M, Healy V, Kealy WF, Hogan J, Doyle CT (1998). Human papillomavirus and mixed epithelial tumorsof the endometrium. Hum Pathol.

